# Exploration of Multiverse Activities of Endocannabinoids in Biological Systems

**DOI:** 10.3390/ijms23105734

**Published:** 2022-05-20

**Authors:** Tapan Behl, Rashita Makkar, Aayush Sehgal, Sukhbir Singh, Hafiz A. Makeen, Mohammed Albratty, Hassan A. Alhazmi, Abdulkarim M. Meraya, Simona Bungau

**Affiliations:** 1 Chitkara College of Pharmacy, Chitkara University, Rajpura 140401, India; rashitamakker32@gmail.com (R.M.); aayushsehgal00@gmail.com (A.S.); sukhbir.singh@chitkara.edu.in (S.S.); 2 Pharmacy Practice Research Unit, Clinical Pharmacy Department of College of Pharmacy, Jazan University, Jazan 45142, Saudi Arabia; hafiz@jazanu.edu.sa (H.A.M.); ameraya@jazanu.edu.sa (A.M.M.); 3 Department of Pharmaceutical Chemistry and Pharmacognosy, College of Pharmacy, Jazan University, Jazan 45142, Saudi Arabia; malbratty@jazanu.edu.sa; 4 Department of Pharmaceutcal Chemistry, College of Pharmacy, Jazan University, Jazan 45142, Saudi Arabia; hasalhazmi@gmail.com; 5 Substance Abuse and Toxicology Research Center, Jazan University, Jazan 45142, Saudi Arabia; 6 Department of Pharmacy, Faculty of Medicine and Pharmacy, University of Oradea, 410028 Oradea, Romania; 7 Doctoral School of Biomedical Sciences, University of Oradea, 410087 Oradea, Romania

**Keywords:** endocannabinoid, receptor, neurotransmitter, gastrointestinal, immunological, cardiovascular

## Abstract

Over the last 25 years, the human endocannabinoid system (ECS) has come into the limelight as an imperative neuro-modulatory system. It is mainly comprised of endogenous cannabinoid (endocannabinoid), cannabinoid receptors and the associated enzymes accountable for its synthesis and deterioration. The ECS plays a proven role in the management of several neurological, cardiovascular, immunological, and other relevant chronic conditions. Endocannabinoid or endogenous cannabinoid are endogenous lipid molecules which connect with cannabinoid receptors and impose a fashionable impact on the behavior and physiological processes of the individual. Arachidonoyl ethanolamide or Anandamide and 2-arachidonoyl glycerol or 2-AG were the endocannabinoid molecules that were first characterized and discovered. The presence of lipid membranes in the precursor molecules is the characteristic feature of endocannabinoids. The endocannabinoids are released upon rapid enzymatic reactions into the extracellular space via activation through G-protein coupled receptors, which is contradictory to other neurotransmitter that are synthesized beforehand, and stock up into the synaptic vesicles. The current review highlights the functioning, synthesis, and degradation of endocannabinoid, and explains its functioning in biological systems.

## 1. Introduction

Cannabinoids have, for the last 40 years, been considered to be molecules or substances which are responsible for the psychoactive properties of marijuana and other associated derivatives of cannabis sativa. Its other medicinal properties are also under exploration. The endogenous cannabinoid system is an omnipresent lipid signaling pathway which performs and controls numerous physiological roles within the body and appeared early in its evolution. Cannabis-like molecules or principal endocannabinoid compounds are small functional molecules which have been isolated from arachidonic acid, a membrane layer, and have been named anandamide (arachidonoylethanolamide) and 2-arachidonoylgylcerol [1]. These molecules are directly linked with a class of G-protein coupled receptor (GPCR). The CB1 endocannabinoid receptor has been shown to be extensively distributed in the brain. It controls brain functions, including emotional, motor and cognitive behaviors, and maintains brain homeostasis. The endocannabinoid system is also an imperative modulator of the immune system, autonomic nervous system and micro circulation outside the brain [2]. These molecules are released from the lipid precursors in a receptor-dependent manner and serve as a retrograde messenger of signaling in the synapses of GABAergic and glutamatergic neuronal cells. They also modulate the postsynaptic transmission by associating with dopamine and other neurotransmitters [3]. The endocannabinoids, with the help of a specific update system, are transported into the cells and deteriorate under the presence of the enzymes’ fatty acid amide hydrolase and mono acylglycerol lipase. Recent pharmacological advances encouraged the production of agonist and antagonist of cannabinoid receptors, anandamide uptake and endocannabinoid degradation inhibitors. Such new technological interventions have led to the thorough exploration of suitable physiological functions exhibited by the endocannabinoids, and exposed newer strategies to cure several neurological, gastrointestinal, immunological, cardiovascular diseases, and cancer [4]. Extensive research has been performed to evaluate the activity and structural elucidation of natural constituents derived from cannabis, also known as cannabinoids, which has led to the development of synthetic compounds that are highly potent and stereoselective in nature, and control major physiological functions of the body. Upon their breakthrough, cannabinoid receptors lead to rapid recognition of lipid transmitters upon availability of highly selective and potent cannabimimetic namely arachidonoylethanolamide (AEA), also known as anandamide and 2-arachidonoylglycerol (2-AG) which serves as a ligand for CB1 receptors [5]. The endocannabinoids possessed pharmacological activity which was similar to that of the synthetic cannabimimetic compounds. Over 3500 scientific reports since the discovery of these molecules have been comprehensively published. These have covered the main physiological aspects of endocannabinoid system. The endocannabinoid system has emerged as a crucial modulator of physiological functioning of the human body, not just restricted to the central nervous system (CNS), but also equally operational in the human endocrine, autonomic nervous system, immunological, reproductive, gastrointestinal, and micro circulation [6,7]. The current review highlights the various physiological roles of the ECS in the functioning of different organ systems of the human body.

### 1.1. A Brief History of Endocannabinoid System

In 1988, Vincenzo Di Marzo, a globally renowned cannabinoid scientist, established the role of endocannabinoid in human physiology and certain bodily functions including mood alteration, sleep, memory, perception to pain and also in maintaining balance in the body. The endocannabinoid system is named after its source of discovery, the Cannabis plant, and is a prominent physiological system that is indulged in establishing and maintaining human health. The system comprises of lipid based retrograde neurotransmitters called endocannabinoid which conjugate with the cannabinoid CB1 and CB2 receptors and induce physiological and molecular responses in the body. The cannabinoid receptors are present throughout the body, including in brain, immune cells, connective tissue organs, and different glands and tissues of the body [8]. The functioning of endocannabinoid neurotransmitter depends upon the presence of its receptors in different tissues, which further leads to stimulation and activation of different molecular pathways and thereby also helps to attain homeostasis and hence can be targeted in treatment or management of a wide array of chronic diseases [9]. The endocannabinoid system was discovered less than 30 years ago, and its role and functioning have been found to be astonishingly widespread in human physiology. A renowned psychoactive compound, namely Tetrahydrocannabinol (THC), was first identified and isolated in the year 1964 by Dr Raphael Mechoulam, who is celebrated as the godfather of cannabis research. Mechoulam also isolated cannabidiol (CBD), a non-psychotropic cannabinoid which possesses neuroprotective and antioxidant properties. Since these cannabinoids naturally occur from plants, they are also named phyto cannabinoids. Isolation of phyto cannabinoids was the primary step and critical event in the discovery of the endocannabinoid system. A molecular biologist, namely Lisa Matsuda, in association with her colleagues at the National Institute of Mental Health in the year 1990, identified a receptor sensitive to THC in the brains of laboratory rats and led to characterization of endocannabinoid system [10]. The endocannabinoids or cannabinoids which are produced in the body by the brain naturally successfully bind to the cannabinoid receptors which are present on the target cells throughout the body and trigger cellular responses which are amplified or diminished as metabolic enzymes destroy or make more endocannabinoids. The endocannabinoids produce a diverse range of activities, from anti-inflammatory to conditions of euphoria [11]. These discoveries led to an explosion of research in the investigation of ECS. The system implicated its role in several physiological functions, which led to the determination of valuable knowledge about the biochemistry, pharmacology and clinical effects of endocannabinoids. They have also been found to serve a protective role in several medical ailments. 

### 1.2. Biochemistry, Production and Secretion of Endocannabinoids

The endocannabinoids were discovered to be the products of arachidonic acid, a molecule similar to other associated transmitters of lipid like leukotrienes, prostaglandins, and eicosanoids. The endocannabinoids are mainly arachidonic acid derivatives and are conjugated with ethanolamine or glycerol and are present in plasma, the brain, and peripheral tissues [12]. Accumulation of anandamide is 200 times lower in the brain compared to the concentration of 2-arachidonolylglycerol (2-AG). The 2-AG monoglyceride is a metabolic intermediary in the process of breakdown of lipid while anandamide is a byproduct of phospholipid membrane cleavage. Upon stimulation of receptor or depolarization, the levels of anandamide can increase up to 5 to 12 times in a time-limited manner [13,14]. The synthesis and release of anandamide occurs through different pathways. The precursor of phospholipid membrane N-arachidonoyl-phosphatidylethanolamine (NAPE), which is mainly produced by N-acyltransferase (NAT) enzyme, leads, upon cleavage, to the formation of anandamide. NAT in the presence of calcium and cyclic adenosine monophosphate (cAMP) catalyzes the transportation of arachidonic acid to the head phosphatidylethanolamine group from phosphatidylcholine. Calcium ions and cAMP significantly enhance the action of the NAT enzyme by phosphorylating cAMP-dependent action of protein kinase A [15]. Specific phospholipase D (PLD) are also involved in catalyzing the release of anandamide from NAPE which had been recently cloned. It is mostly present in the brain, kidney, and testes. Since oleoyl and palmitoyl acids are incorporated in the sn-1 position of NAT, there are preferable chances of synthesis of PEA and OEA from NAT rather than anandamide. The production and discharge of anandamide and 2-AG are entirely different. 2-AG are monoglycerides and their arrangement is related to the breakdown of receptor medicated activation of triacylglycerol by phosphatidylinositol-specific phospholipase C (PLC). An increase in production of 2-AG has been noted upon the stimulation of metabotropic receptors linked with diacylglycerol lipase route and PLC. The formation of 2-AG is dependent upon the concentration of calcium ions while its modulation is independent to the production and secretion of anandamide [16,17].

### 1.3. Endocannabinoids Derived from Eicosapentaenoic and Docosahexaenoic Acids

The endocannabinoid molecules are endogenously also synthesized from omega-3 and omega-6 polyunsaturated fatty acids (PUFA). They are the products of fats obtained from the diet, and any changes in the dietary intake can modulate their levels in the body, which results in the displacement of arachidonic acid from the membranes of cells by eicosapentaenoic acid (EPA) and docosahexaenoic acid (DHA) based endocannabinoids, thereby reducing the levels of 2-AG and anandamide [18]. The research depicting the effects of saturated fats on the endocannabinoid system are much less, and are exceptionally limited to stearic and palmitic acid. However, a study in starved mice has shown a significant reduction in their diet intake upon intravenous administration of stearoyl ethanolamide. Also, the administration of omega-3 fatty acids leads to a higher proportion of omega-3 endocannabinoids, which presents several physiological actions in the body. The increased production of eicosapentaenoyl ethanolamide (EPEA) and docosahexaenoyl ethanolamide (DHEA) from its precursor molecules eicosapentaenoic acid and docosahexaenoic acid respectively can be achieved in lieu of its ability to displace arachidonic acid from the cellular phospholipid membrane. DHEA was firstly isolated from the bovine retina and presents 3 hypotheses for its biosynthesis. The first mechanism involves its synthesis by cleavage of N-docosahexanoyl-phosphatidylethanolamine (NDHPE) from its phospholipid backbone by enzyme phospholipase D2, which is similar to the synthesis of anandamide. Hydrolysis of NDHPE by α/β-hydrolase 4 enzyme and condensation of DHA and ethanolamine directly can also lead to synthesis of DHEA. The synthesized EPEA and DHEA present anti-inflammatory activity by decreasing the production of adipocyte interleukin-6 and monocyte chemotactic protein-1 in mice. The synthesized DHEA was found to be 8–22.5 times less potent and the EPEA had 4–16.5 times weaker binding association to CB1 receptors compared to anandamide, and DHEA were endogenously found in retina and brain. The DHEA and EPEA have also been detected in human milk and have been noted to play a significant role in the management of inflammation, modulation of dietary intake, cancer and central nervous system, thereby urging a need for further investigation of their physiological and pharmacological properties [19].

### 1.4. Physiological Endocannabinoid-like Molecules

Anandamide has been known to be a potent endocannabinoid molecule for a long time, and belongs to a category of naturally occurring molecules. N-palmitoylethanolamine (PEA) is one of its members, and has been reported in humans for more than 50 years. The physiological roles of PEA are being explored through extensive research. PEA and other related naturally occurring molecules share degradative mechanisms with endocannabinoid molecules and target the cannabinoid receptors competitively by inhibiting their hydrolysis or by modulating the binding site of receptor allosterically, and are also known as endocannabinoid-like molecules. Another agent with noted activity of suppression of appetite namely N-oleolylethanolamine (OEA) has also been listed as an endocannabinoid-like molecule. The biological activity of OEA engages the activation of PPAR α and transient receptor potential vanilloid type 1 (TRPV1), which is one of the renowned non-CB receptor targets for endocannabinoids and is also activated by both anandamide and 2-AG. OEA presents the highest affinity towards PPAR α in comparison to all endocannabinoid like compounds. Other endocannabinoid like compounds, namely 2-oleoylglycerol (2-OG), PEA and OEA, have a tendency to activate GPR119, which is a GPCR expressed predominantly in pancreas of rats and humans indicating the action of OEA on dietary intake and presented hypophagic effects in mice. PEA and OEA molecules have also been found to engage additional receptors such as GPR55 at high concentrations. Another naturally occurring endocannabinoid like molecule, namely N-stearoylethanolamine (SAE), has been also found to be a controller of cell growth with additional anti-inflammatory and immunomodulatory activities. SEA in combination with PEA plays an anti-nociceptive role in humans and also presents anorexia effects. The concentrations of PEA, SEA, OEA and 2-OG endogenously can be affected by the intake of different diet regimes and can impact differently the brain tissues compared to peripheral tissues. They are being extensively explored through advanced research to elucidate their actual physiological relevance in the human body. PEA is currently marketed for management of pelvic and neuropathic pain due to its lack of adverse effects centrally at CB1 receptors [20]. Less information is available regarding the endocannabinoid like molecules and these are being extensively studied since, although they have poor affinity towards cannabinoid receptors, they can be included therapeutically to produce significant clinical effects.

### 1.5. Uptake and Degradation of Endocannabinoids

**Figure 1:**
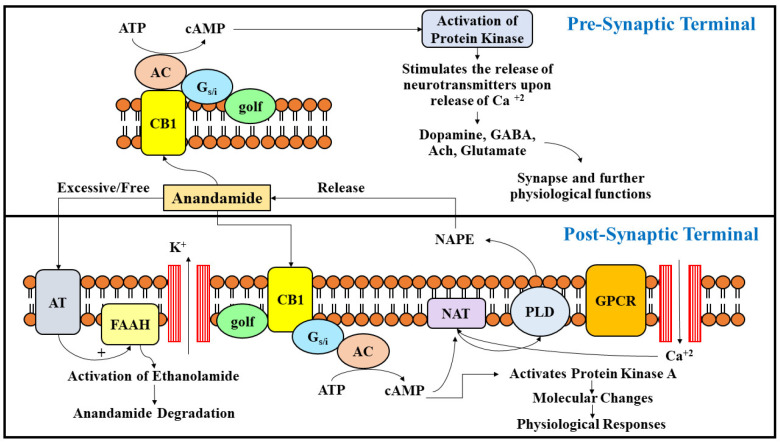
The figure presents a general overview on the synthesis, release, uptake, and degradation of endocannabinoid Anandamide. NAPE is a precursor of lipid, which is the source of release of anandamide upon activation of phospholipase D and further stimulation of GPCR. NAT is a catalytic enzyme which hastens the biosynthesis of NAPE upon release of cAMP and calcium ions. Upon activation of cannabinoid receptors at presynaptic sites, they are released into synapse and further initiate the release of several neurotransmitters which control and modulate various physiological functions in the body. The excitation of neurons is also regulated upon nodulation of the potassium channel, and regulates the release of protein kinases. The action of anandamide is terminated by its cellular uptake via anandamide transporter or its degradation upon activity of FAAH enzyme.

The signaling of endocannabinoids is mainly ceased by a two-step process which includes its transportation into cells which is further hydrolyzed by specific enzymatic systems. Most of the steps are crucial and ensure a tightened monitoring of endocannabinoid concentrations in tissues, and promptly eliminate the substances that initiate signaling. The uptake of endocannabinoids is mediated by a transporter which is extensively dispensed throughout the brain [21,22]. The transporter works in a fashion similar to other lipid carriers and is an elusive molecule which facilitates the uptake of 2-AG and anandamide, both based on their energies. The transporter of anandamide is saturable and displays a specificity for its substrate. Its activity can be blocked by specific drugs [23]. The potential coupling of transporters and degradation substances have been a major issue of debate as the energy that is required for the process of its uptake is achieved by combining it with anandamide enzymatic hydrolysis [24]. Endocannabinoids are degraded by the action of two specific enzyme systems, namely the fatty acid amide hydrolase (FAAH) and the monoacylglyceride lipase (MAGL). FAAH belongs to a family of serine-hydrolase and is a membrane enzyme which is broadly allocated within the human body with levels greater in the brain and liver. Several fatty acid amides involving acylethanolamides like oleamide, sleep factor, and anandamide, are mainly degraded by the activity of FAAH. 2-AG can also be inactivated by the enzyme FAAH but the major enzyme accountable for its inactivation is MAGL [25]. The DHEA can be metabolized by cyclooxygenase, lipooxygenase and CYP450 enzymes, thereby forming endocannabinoid metabolites, which were found in the brain, hemoglobin and polymorphonuclear leukocytes. However, EPEA was metabolized by enzyme CYP450 and produced corresponding epoxide metabolites, which were identified in rat tissues (Figure 1).

### 1.6. Cannabinoid Receptors

The cannabinoid receptors are mainly of two types, and belong to the super family of GPCR. The primary receptor, CB1, is majorly found in the peripheral and central neuronal cells terminus, glial cells, reproductive system (mainly testes), micro circulation, and other major glands of human body [26]. The cannabinoid CB2 receptors were initially found in multiple lymphoid organs and were highly expressed in the B lymphocytes, moderately expressed in polymorphonuclear neutrophils and monocytes and were least expressed in the T-lymphocytes. The expression of cannabinoid receptors during the development of the brain is the most fascinating aspect as they can control differentiation of cells and are present in tumor cells derived from the main epithelia and glial cells [27,28]. CB1 cannabinoid receptor demonstrates distinctive pharmacological properties and comprises a 97–99% amino acid sequence identity depicting its ancient role in signaling of several molecular pathways in vertebrates and several invertebrate phyla. They impart a remarkable role in human system physiology [29,30]. The CB1 receptors are also highly expressed in the brain and are the most abundantly expressed G protein coupled receptors with an intensity 10 to 50 times more than classic neurotransmitters like opioid receptors and even dopamine. The efficiency of CB1 receptors is comparatively low. Cannabinoid receptor 1 (CB1) and cannabinoid receptor 2 (CB2) in combination with other molecular receptors like peroxisome proliferators activator receptor (PPARs) and transient receptor potential channels (TRP) regulate the functioning of endocannabinoid in the human body. Upon their activation, the stimulation of adenylyl cyclase and other associated voltage-dependent calcium channels is inhibited, which further triggers initiation of mitogen activated protein (MAP) kinase, and rectifies potassium channels inwardly [31]. Therefore, both these receptors exert a functional activity in cellular physiology, including functioning of the synapse, genetic transcription, etc.

The central nervous system has plentiful supply of CB1 receptors, particularly in the region of the cortex, basal ganglia, cerebellum and hippocampus. These receptors are particularly present on the axonal terminals and on the pre-terminal segments of the axon and spare the active zone on the neuron cell. The CB1 receptors in the hippocampus and cortex are rich in cholecsytokinin (CCK)-positive inter-neurons and are also extensively articulated at lower levels in the glutamatergic neurons. In addition, the medium spiny neurons of the ventral and dorsal striatum region contain an abundant number of CB1 receptors [32]. The expression of CB1 receptors is on a higher side in the axons, which directly connect with the globus pallidus region and further head towards the substantia nigra region. CB1 receptors in the cerebellum are found in parallel and climbing fibers, and in the basket cells. Apart from the active presence of CB1 receptors in the neuronal cells, these receptors are also found to be functionally relevant in the glial cells and many other independent groups [33,34].

Accumulation of genetic and animal model experimentation data suggested a significant role of CB2 receptors in schizophrenia; however, the presence of CB2 receptors whether in neuronal, microglia or neuro-developmental neurons, is still under evaluation (Table 1).

### 1.7. Endocannabinoids and Their Pharmacology

Research efforts have intensified in the past two decades, especially since the discovery of the cannabinoid CB1 receptor and anandamide, leading to the identification of several drugs which directly interact with the chief elements of the endogenous cannabinoid system [35]. Several drugs have been identified to date which directly link with these receptors as an agonist or antagonist, and obstruct the transportation of the endocannabinoid system, also impeding FAAH activity. However, drugs which specifically inhibit the enzymes NAT, PLD, MAGL, etc., are still being discovered. Before the radioligand cannabinoid receptors were available, the in-vitro bioassays instigated constraint on the production of forskolin-stimulated cAMP, thereby blocking the contraction of the isolated smooth muscle [36]. The smooth muscles mostly used in bioassay of cannabinoids were isolated from the myenteric plexus and vas deferens of the mouse, and even included small intestine isolated from Guinea pigs. The bioassays performed were highly sensitive and relied on the capability of agonist of cannabinoid receptors to interact with CB1 and prevent the invoked contractions of the muscle. General behavioral tests were also performed to assess the locomotion and analgesic activity of the CB1 receptors via in vivo bioassay methods. It was concluded that analgesia, immobility, hypothermia, and catalepsy were the four effects which were classically considered as signature of cannabimimetic activity [37,38]. To study and evaluate the activity of specific compounds, several mouse models are being used which comprise knocked out cannabinoid receptors and FAAH enzyme. The primary purpose of the research is to design and develop newer safer drugs which mimic the signaling processes of cannabinoid receptors as they were formerly mediated by 2-AG and anandamide, and could boost the activity of CB1 receptors when required [39]. In conditions with enhanced endocannabinoid signaling, the antagonism of cannabinoid receptors might be a suitable approach. The action of endocannabinoids can also be enhanced by inhibiting its transportation and degradation, and this therefore indicates the relevance of exploration and rational use of therapeutic drugs which strategize the functioning of endocannabinoid signaling in humans and thereby helps to prevent several disorders. The signaling of anandamide can be enhanced in conditions of stress by counteracting the degradation of anandamide which can be used to overcome the deficiency of this molecule [40,41]. Examples include ACEA and SR141716A, which are potent the agonist and antagonist of CB1 receptors respectively and HU-308 and SR 144, 528 act as agonist and antagonist for CB2 receptors respectively. OL-135 was identified as a reversible FAAH enzyme inhibitor while URB 597 acts as an irreversible inhibitor of the enzyme. The endocannabinoid molecules, apart from their interaction with cannabinoid receptors, also extend beyond their biological attributes, including oxidation of anandamide and 2-AG by cyclooxygenase-2 enzyme, lipooxygenases and derivatives of prostaglandins. The biological properties of prostaglandin derivatives are quite different from the parent substrates. For instance, the prostaglandin ethanolamide like prostamides and prostaglandin glyceryl esters are not only pharmacologically different from their parent endocannabinoid molecules but also vary from their corresponding prostaglandin molecules acidic in nature. 2-AG and anandamide can also be oxidized by P450 enzymes and do not specifically represent the inactivation of endocannabinoids but alter and mimic the activity of endocannabinoids through different pharmacological pathways. The oxidation of endocannabinoid molecules and production of their metabolites are highly complex compounds and are being explored for their therapeutic potential [42].

## 2. Physiological Role of Endocannabinoids in Human Gastrointestinal System

The role of CB1 receptors was suggested in regulating the contractility in the gastrointestinal tract more than a decade before they were discovered. The motility of the gastrointestinal tract in mice was inhibited by the oral consumption of the primary psychoactive cannabis molecule, namely Δ-tetrahydrocannabinol (THC), thereby adjusting its principal protective role in the human gastrointestinal system. The CB1 receptors are located in the vagal sensory terminals and neurons of the enteric nervous system of the human gastrointestinal tract, while CB2 receptors were found in immune cells [43]. CB1 receptors activate and initiate several functions of the gastrointestinal tract such as secretion of gastric juices, motility of intestine, and emptying of stomach. This implies a significant role of endocannabinoids in the gastrointestinal tract and acts as a potent gastroprotective agent [44]. The CB1 Receptors prominently play a dominant role in the human gastrointestinal system compared to other compounds isolated from Cannabis sativa extract. 2-AG was initially extracted from the intestine of canine in 1995, and sometime later anandamide was also obtained from the tissues of mice small intestine with simultaneous presence of the FAAH enzyme [45]. The myenteric plexus also showed the presence of CB1 receptors while CB2 receptors were mostly found in the whole segment region of the intestine indicating the presence of the latter mostly in the blood cells [46]. The proteins for CB1 receptors in the gut of the mouse were determined through immunoblotting various segments of the gut. CB1 receptors are mostly localized with the acetylcholine transferase enzyme in the gut, which shows the presence of cholinergic neurons and further encourages their active role as potent inhibitors of motility of intestines and gastric secretions, mainly by preventing the neurotransmission through cholinergic neurons in the gastrointestinal tract [47]. The CB1 receptors were also found to be partially localized with immunoreactive substance P in the neurons of the intestine, thereby indicating they can help or modulate reaction on the gastrointestinal tract of the humans, and can alter the sensory motor function of intestines [48].

### 2.1. Motility

The CB1 receptors are inhibited by the endogenous and synthetic cannabinoids in a dependent fashion and electrically evoke contractions in the small intestine isolated from guinea pigs, thereby modifying the intestinal motility upon their exposure. Anandamide was also found to inhibit the release of acetylcholine through generation of electrical signals via stimulation of CB1 receptors and simultaneously acts on the TRPV1 receptors. The functional activity of anandamide in modulating the motility of the intestine is being explored by investigating its physiological and pathophysiological states. The inhibitors of FAAH enzyme have demonstrated reduced motility of intestines which can be overcome by administering antagonists of CB1 receptors. The hypomotility of the intestine, which is a typical characteristic of paralytic ileus, is also thought to occur due to enhanced levels and expression of anandamide and CB1 receptors, which demonstrates that the acetic acid induced ileus can be elevated by SR1415716A, a CB1 receptor antagonist and can be worsened by inhibiting the cellular uptake of anandamide by VDM11 [49,50].

### 2.2. Gastric Emptying and Intestinal Motility

The rate of gastric emptying and motility of the small intestines in humans and rodents was slowed upon administration of Δ9-THC intravenously. The cannabinol and Δ9-THC were also noted to reduce the motility of intestines in mice, but, relatively, were not particularly effective in emptying the gastric contents. Upon administration of CB1 agonists, namely CP55,940 and WIN55,212-2, and blocking the activity of SR141716A, a selective antagonist of CB1 receptor the above results were confirmed. The contraction of pyloric muscles and intragastric pressure was also found to be decreased upon intravenous administration of Δ9-THC. The ganglionic blockers, vagotomy and antagonists of CB1 receptors like SR141716 significantly modulated the changes in the motor functioning of the gastrointestinal tract. Apart from CB1 receptors, the role of CB2 receptors has also been established in controlling the mortality of the gastrointestinal tract and gastric emptying [43,51]. The gastrointestinal motility was also increased upon treatment with lipopolysaccharides. The CB2 agonists were successfully able to control the increased mortality in such scenarios in a dose-dependent manner compared to the CB1 agonist, and also prevented the blockage of CB2 agonist by the activity of selective CB2 antagonist. To re-establish the normal gastrointestinal motility upon stimulation of inflammatory parameters, CB2 agonist receptors can be successfully employed in response to the lipopolysaccharides and modulate the gastrointestinal functioning [52]. A decrease in the levels of 2-AG endocannabinoids in the body mass and brain of humans and animals has been observed upon supplementation of DHA and EPA which reduced weight gain in the mice and further prevented the development of obesity by increasing β-oxidation, in turn decreasing the stimulation of cannabinoid receptor due to reduced production of anandamide and 2-AG and therefore decreasing appetite and dietary intake.

### 2.3. Secretion of Gastric Acid

The role of endocannabinoids has been established in regulating the secretion of acid from gastric mucosa through several studies. Bioassay studies performed on the stomach isolated from rats showed that high doses of Δ9-THC successfully counteracted the secretion of gastric acid in response to histamine activation. The gastric secretions in rats mainly induced by pentagastrin were successfully blocked upon administration of WIN55,212-2 and HU-210 through peripheral mechanisms. These effects were mainly inhibited by the antagonists of CB1 receptor, which demonstrated the anti-gastric acid secretary effects of cannabinoids by preventing the activation of CB1 receptors which are mainly located on the cholinergic pathways of preganglionic and postganglionic sites. Excessive gastric acid production causes conditions like peptic ulcers and gastritis in the duodenum region of intestine and stomach. The vagal efferent pathways comprising of CB1 receptors are prominently involved in reducing the production of gastric acid in the stomach and prevent such gastrointestinal disorders [53]. Upon activation, the CB1 agonists reduce the release of histamine from enterochromaffin cells, thereby indirectly mediating a lower secretion of gastric acid from the parietal cells due to deficiency of histamine. This mechanism can be marked as a possible pathway which mediates a reduction in secretion of gastric acid upon activation of CB1 receptors in animal models to report anti-ulcer activity in rats. The clinical use of endocannabinoids still remains speculative, although a decrease in secretion of gastric acid upon consumption of cannabis has been observed and an exact evaluation of the same is still not clear. Apart from its beneficial role in the treatment and prevention of peptic ulcers, the cannabinoids can also be indulged in management of gastroesophageal reflux disease [54].

### 2.4. Food Intake Alteration

The endocannabinoid system has also been found through various studies to modulate the intake of food through activity of cannabinoid receptors located in the brain and in peripheral tissues. The CB1 receptors, upon selectively interacting with the capsaicin sensitive sensory terminals, can modulate the intake of food and thereby present an unexpected role of endocannabinoids in control and monitoring of diet, and can regulate feeding [55,56]. The endocannabinoids are being explored for their pathophysiology in the gastrointestinal tract, and are being characterized as therapeutic treatment in curing several gastrointestinal tract (GIT)-associated ailments. With an increase of resources and technology, the evident role of cannabinoid is being exposed in the treatment of a variety of gastrointestinal dysfunction in humans. Several patient reports and anecdotal data have outlined the role of endocannabinoid extracts in the management of abdominal cramps and diarrhea [57,58].

### 2.5. Emesis

**Figure 2:**
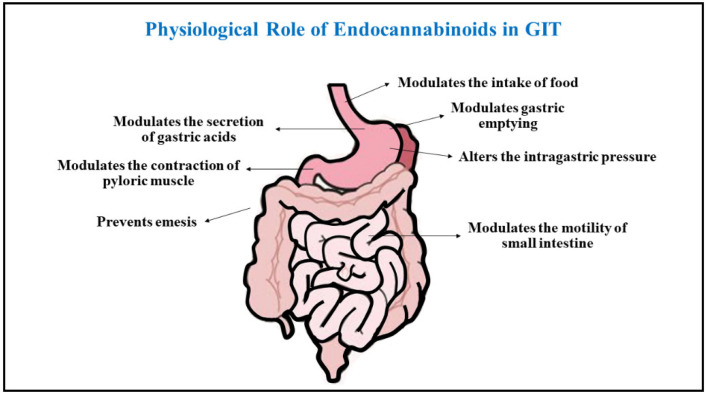
The figure describes a collective overview on physiological roles attained by endocannabinoids in the management of gastrointestinal complications.

Endocannabinoids possess anti-nauseous and antiemetic effects which were established upon clinical investigation in cancer patients who were subjected to chemo and radiation therapies, and also in patients who received human immune virus (HIV) treatment. Both central and peripheral sites of the patients were involved for activity of endocannabinoid receptors [59,60]. The vagal efferent present in the peripheral sights and the area postrema and dorsal vagal complexes area in the central site are majorly involved as a site of action for the endocannabinoid receptors. It has been found that nausea can be induced due to a delay in the emptying of gastric contents due to cannabinoids, suggesting that the role of central effects is more prominent than the peripheral effects in management of emesis and nausea [61]. To date, emesis and nausea caused by cancer and malignancies have been found to be a proven indication for cannabinoids within the gastrointestinal pathologies, and several clinical trials are being performed to establish the beneficial activity of cannabinoids in the treatment of chemotherapy-induced vomiting and nausea and upgraded successful therapeutic role in the prevention and management of emesis [62] (Figure 2).

## 3. Physiological Role of Endocannabinoids in the Human Cardiovascular System

The cannabinoid receptors have been found to be widely distributed throughout the cardiovascular system. The CB1 receptors are present in the coronary artery, endothelial cells, myocardial cells, and smooth muscle cells besides the sympathetic nerve terminals present on presynaptic site which intervene with the cardiovascular system [63]. The presence of CB2 receptors has also been detected in the human coronary endothelial cells, myocardial cells, and smooth muscle cells of the heart. Endocannabinoids are synthesized in the smooth muscle and endothelial cells of the cardiac tissue, and can be detected in the circulating blood, and thereby play a significant role in regulation and management of the functioning of the cardiovascular system. The animal models who possess knocked out FAAH enzyme fail to show any major changes in the functioning of the cardiovascular system, implicating an evident role of cannabinoid molecules and their receptors in cardiovascular physiology. Mice which have a knocked out FAAH enzyme showed a diminished decline in age-mediated functioning of the cardiac system, thereby making the animals susceptible to atherosclerotic diseases. Studies with mice having knocked out CB1 receptors have shown them to be more vulnerable to conditions like stroke and chronic heart failure, and deficiency of cannabinoid receptors have been detected to be more amenable towards atherosclerosis and cardiomyopathy, indicating a crucial role of the endocannabinoid system in maintaining healthy functioning of the cardiovascular system [64,65].

### 3.1. Endocannabinoids in Modulating Heart Rate and Blood Pressure

A significant action of endocannabinoids has been established on the blood pressure and rate of heart beats of animals selected in several cardiovascular studies, and the responses noted were dependent on the condition of the animal, whether they were conscious or anesthetized, which may be due to alteration in the sympathetic activity of basal levels due to anesthesia as the endocannabinoid also respond by modulating the autonomic nervous system and act through vagal and sympathetic activity [66]. Anandamide has been noted to produce a triphasic response in the anesthetized animals which included a transient decrease in heart rate, also known as bradycardia, increase in blood pressure due to transient pressure responses, and extended phases of hypertension. The initial onset of bradycardia in response to anandamide in mice with knocked out transient receptor potential vanilloid 1 (TRPV 1) is induced by activation of vagal neurons. Anandamide has been characterized to possess an extended response of hypotension in animals that are anaesthetized, and a similar response is absent in mice that are knocked out with CB1 receptors, thereby inhibiting the sympathetic activity on the nerve terminals present at the level of heart and vasculature. CB1 receptors upon activation in arteries inhibit the release of noradrenaline in the mesenterial arterial bed region [67]. A steep decrease in blood pressure accompanied with tachycardia was also noted in anesthetized rats who are administered with 2-AG. The activity of 2-AG is independent of CB1 receptors, unlike anandamide, and majorly involves catalyzation and metabolism of cyclooxygenases to produce other associated vasoactive compounds. Anandamide produces profound bradycardia in conscious rats with a transient condition of hypertension followed by long lasting pressor effects, which are majorly accompanied with renal and mesenteric vascular bed mediated vasoconstriction [68]. It is also accompanied by vasodilator responses mediated to the beta-2 adrenergic receptors. A highly complex hemodynamic effect was noted due to increase in the amount of circulating adrenaline in blood which acted via adrenergic beta-2 receptors and increased sympathetic activity upon activation of CB1 receptors. The endocannabinoid system was discovered to be involved in controlling the blood pressure centrally by associating with the brainstem baroreceptor complexes [69]. One of the termination sites of baroreceptor efferent fibers is the nucleus tractus solitarius which arises from the baroreceptor’s present in arteries and cardiac mechanoreceptors. The CB1 cannabinoid receptors are functionally expressed in the nucleus tractus solitarius region and an anandamide microinjection can prolong the reflex inhibition of the renal sympathetic nerve activity and suggest that inhibition of the GABAergic tone can lead to an increase in sensitivity to baroreflexes. An increase in blood pressure due to phenylephrine can also enhance the concentrations of anandamide in the region of nucleus tractus solitarius and support the physiological relevance of endocannabinoids in controlling and regulating the activity of baroreflexes [70]. A recent study performed in mice with lock down CB1 receptors showed significant conditions of elevated pressure in blood and rate of heart beats in response to irregular breath during sleep and disturbed sleep-awake cycle.

### 3.2. Endocannabinoids and Hypertension

A hypotensive effect has been found to be prolonged upon administration of anandamide in anesthetized rats compared to rats in normotensive conditions. The agonists of CB1 receptors and FAAH enzyme inhibitors normalize the blood pressure and decrease myocardial contractions in anesthetized hypertensive animals [71]. However similar experiments in conscious animals depicted mild responses towards anandamide, even though the animals with hypertension presented bradycardic responses due to activation of CB1 receptors by anandamide, which was missing in normal animals. The expression of CB1 receptors was found to be significantly higher in the aortic endothelium and cardiac tissue of spontaneously hypertension rats as compared to healthy normal control rats, indicating the expression of endocannabinoid system in altering conditions of hypertension [72]. Rimonabant, an antagonist of CB1 receptors, demonstrated marginal effects on the blood pressure in the subjects with normotensive conditions, but a huge reduction in the blood pressure was observed in hypertensive obese and Type 2 diabetic patients, suggesting the activation of endo cannabinoid CB1 receptors could be a therapeutic approach. The circulating levels of endocannabinoids and blood pressure of the individual has shown positive correlation in several studies, which supports the noticeable role of endocannabinoids in managing blood pressure. A study which was performed to correlate the relationship between circulating levels of anandamide and patients with obstructive sleep apnea showed that anandamide posed to be a stronger determinant of blood pressure as compared to obesity, inflammation, severity of sleep apnea resistance to insulin. The mean arterial blood pressure and diastolic pressure in a study comprising of females with depression showed a positive correlation with levels of anandamide and 2-AG in the serum [73].

### 3.3. Endocannabinoids and Shock

Shock is a condition which is mainly characterized by diminished cardiac output, which further reduces the blood pressure of an individual and affects tissue perfusion. The decrease in blood pressure due to hemorrhagic shock can be prevented by administering antagonists of CB1 as per a study performed by Wagner and colleagues in 1997. The researchers stated that synthesis of anandamide and 2-AG by platelets and monocytes was the responsible cause for activation of CB1 receptors in condition of shock. Later, many studies were performed to evaluate the role of CB1 receptors in conditions of cardiogenic and endotoxic shock post episodes of myocardial infarction [74]. Upon central administration of antagonists of the CB1 receptor, desirable effects were not observed, indicating the presence of a peripheral mechanism of action for its activity. The CB1 receptors present in arteries upon activation directly trigger vasodilation and inhibit the release of sympathetic neurotransmitters, which further cause vasodilation and instigates a fall in the blood pressure. The endocannabinoids have been advocated to offer a cardioprotective role in patients with shock, and antagonists to CB1 receptors have successfully decreased mortality in animal models of shock [75].

### 3.4. Endocannabinoids and Arrythmia

**Figure 3:**
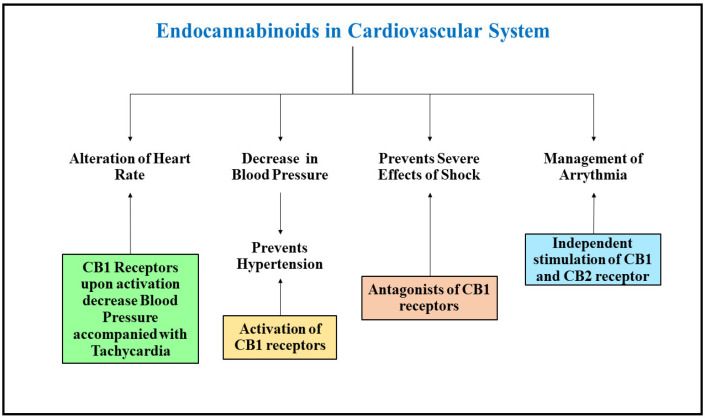
The figure describes the protective role of Endocannabinoids in management of cardiovascular disorders.

Upon activation, the CB2 receptors ominously reduce the prevalence of ventricular arrythmia during occlusion of coronary arteries. The arrhythmias induced by epinephrine is also condensed by the independent stimulation of cannabinoid receptors in response to anandamide release. Recently, both anandamide and 2-AG were shown to be ineffective towards ventricular fibrillation and ischemia, although an antagonist of CB1 receptors did present positive impact despite subsequent ischemic stages. The samples isolated from rabbits showed subsided duration of action potential amplitude upon stimulation of CB1 receptors by anandamide [76]. Upon independent activation of CB1 and CB2 receptors by anandamide, the voltage regulated sodium and calcium channels (L-type) were inhibited, which stimulated the action potential of the rats’ ventricular myocytes [77] (Figure 3).

## 4. Physiological Role of Endocannabinoids in Immune System

**Figure 4:**
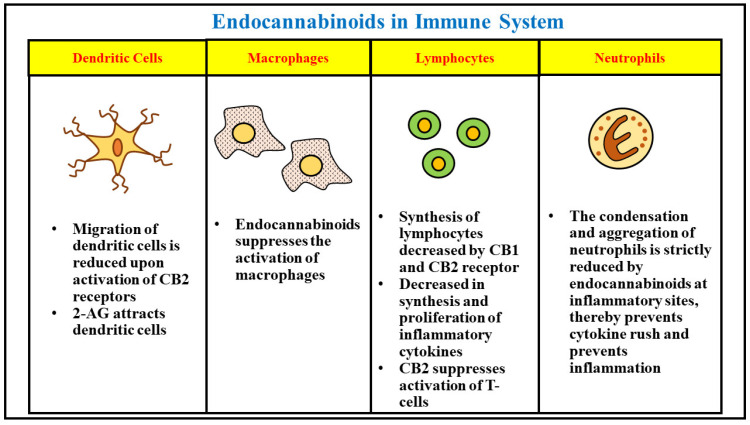
The figure concisely presents the role of endocannabinoids in growth, proliferation, activity and functions of different types of inflammatory cells in the immune system.

The endocannabinoids also play a significant role in controlling the functioning of the immune system and help to maintain immune homeostasis. The immune cells express both the types of endocannabinoid receptors CB1 and CB2, and lead to further secretion and transportation of these molecules and regulate the breakdown mechanisms. The receptors of endocannabinoid are highly expressed in immune B cells, followed by monocytes, natural killer cells, neutrophils polymorphonucleated, CD4 and CD8 lymphocytes. The CB1 receptors facilitate neurobehavioral effects due to their dense expression in the central nervous system [78]. The CB2 receptors are over 10–100 times more manifested in the immune cells compared to the CB1 receptors, indicating the role of numerous endocannabinoid compounds in the regulation of immune responses [79]. The concentration of 2-AG endocannabinoids was found to be higher in the plasma of blood compared to plasma of the bone marrow cells. A proven role of CB2 receptors has been found in retaining the immature B cells of the bone marrow and are also involved in inhibiting the recovery of lymphocytes post transplantation of bone marrow [80]. CB2 receptors present on neutrophils mediate the anti-inflammatory actions of released endocannabinoids from the P-glycoprotein. A deficiency of CB2 receptors led to acute exacerbation of the mobilization of neutrophils to the inflammation sites. The CB1 and CB2 receptors also mediate the regulation of other associated innate immune cells, such as macrophages, monocytes, and microglial cells, where they engage in augmenting immunosuppressive activity. The activity of macrophages associated with tumors is also believed to be inhibited by CB2 receptors. The migration of signaling through dendritic cells is also believed to be suppressed under the presence of CB2 receptors by inhibiting the expression of matrix metalloproteinase 9. Apart from their role in regulating the immune cells, the endocannabinoid system also regulates adaptive immunity of an individual. The expression of CB2 receptors in T cells in comparison to immune cells is seen to a lesser extent; however, upon stimulation, T cells can up-regulate the manifestation of CB2 receptors and can also cause the expression of CB1 receptors. The elementary cytokine implicated in the activity and differentiation of T cells is interleukin 2, which is secreted upon the activation of natural killer cells and T-cells. The synthesis of interleukin 2 is condensed upon activation of both CB1 and CB2 receptors. Anandamide presented a significant role in functioning of mature B cells and induced a dose-dependent immunosuppression. In addition, activation of 2-AG by T-cells and B-cells successfully modulated inflammation, and immunity induced by vaccination was also reduced upon legation of CB2 receptors. Hence, altogether the endocannabinoid system can be considered to play a significant part in modulation as well as functioning of the immunological system. Inflammation, moderate in intensity, plays a protective role against several infectious diseases and provides long term adaptive immunity towards selective microorganisms [81]. Nevertheless, uncontrolled or chronic inflammation rising due to a hypersensitive immune system can result in incessant tissue damage. Patients suffering with autoimmune diseases take medications to benefit from their activated immune system and trim down inflammation. Conversely, patients with poor immunity, such as those suffering from cancer, aim to uplift their immune system to prevent tumors. Hence, endocannabinoid-based treatments can be effective in the management of several immune-related diseases (Figure 4).

### 4.1. Endocannabinoids and Infectious Diseases

The major role of our immune system is to protect the host from foreign antigens and pathogens. The reactive role of endocannabinoids against infectious pathogens and antigens has been established for over 40 years. The combination of lipopolysaccharides with THC has been found to be extremely toxic in mice, and administration of THC showed lethal activity to heat and kill bacteria. In addition, resistance to several other pathogens including listeria and herpes simplex virus was also demonstrated [82,83,84]. This led to the established role of the endocannabinoid system in inducing immune reactions upon exposure to bacterial pathogens, and the presence of specific CB2 receptors were found upon exposure to viral pathogens. The pathogenesis of several bacterial, viral, and microbial diseases was also controlled upon administration of cannabinoids. The oral consumption of cannabis was also found to increase the rate of survival of mice infected with malaria. A study also revealed that administering endocannabinoids in increased levels was helpful in treating mice infected with helminths in intestine and lungs, presenting their immune response at elevated doses combined with post infection immunity [85]. The clinical anti-infective role of cannabinoids can be ascribed to its anti-inflammatory property upon exposure to certain pathogens.

### 4.2. Endocannabinoids and Cancer

The analgesic and anti-emetic effects of endocannabinoids have led to their administration in cancer patients as palliative medicine. The anti-tumor property of several endocannabinoids has also been suggested; since most of the studies were done as in vitro or in immune competent animal models, the data from humans is still hearsay [86,87,88]. The various types of tumors indicated the expression of large diversity of endocannabinoid related molecules, leading to the development of specific cannabinoid-based treatments that are highly effective in cancer patients. The microenvironment of a tumor is a complex ecosystem and comprises of fibroblasts, cytokines, hormones, immune cells, and several other factors, which are directly involved in the progression of cancer [88]. The progression and malignancy of cancer are directly attributed to the immune responses in any individual. The era of management and cure of cancer has been revolutionized recently due to the development and administration of immunotherapy, which was successful to restore immune responses that were deficient in tumor cells, thereby altering the expression of immune cells against cancer. To present personalized therapeutic treatments, it is essential to understand the effect of cannabinoid-based treatments against the pathology of cancer to induce immunity as part of the development of future therapies [89].

### 4.3. Endocannabinoids and Autoimmune Diseases

Several preclinical studies performed in patients with autoimmune diseases established the clinical application of cannabinoid substances in immune-mediated disorders. The cannabinoids were evaluated to successfully generate a balance between the regulatory T-cells and inflammatory Th17, indicating the presence of a regulatory phenotype. The clinical action of endocannabinoids was studied by administering these molecules in various autoimmune and inflammatory disorders. Rheumatoid arthritis is the dominant autoimmune disease and present as a major cause of disability globally, leading to increased inflammation in synovial joints followed by pain and destruction of joint cartilage. The endocannabinoid treatment was found to be successful in selectively blocking the arthritic progression in a collagen-induced arthritis animal model. The conditions of synovitis and joint destruction were also found to be attenuated in collagen-induced arthritic mice upon activation and stimulation of CB2 receptors. The elevation of endocannabinoids upon administration of FAAH inhibitors was also found to be helpful in alleviating symptoms in murine arthritic models. The endocannabinoids anandamide and 2-AG besides an increased levels of FAAH were detected in the synovial fluid of a patient suffering with osteoarthritis and rheumatoid arthritis, indicating their application in management of the diseases [90]. Patients suffering with multiple sclerosis were also clinically examined upon administration of cannabinoid-based treatments. An increased level of anandamide was found in the serum of multiple sclerosis patients compared to healthy patients, and indicated their protective role in management of the disease [91]. The multiple sclerosis patients were observed to comprise of significantly improved muscle spasticity. The cannabinoid receptors and endocannabinoid molecules were also found to possess significant activity in management of similar gastrointestinal inflammatory disorders such as inflammatory bowel disease, ulcerative colitis and Crohn’s disease, etc. The endocannabinoid molecules and receptors upregulated inflammation and stimulated the attenuation of murine colitis while their antagonist successfully reversed the anti-inflammatory effects [92].

## 5. Physiological Role of Endocannabinoids in Human Brain

The role of cannabinoid molecules has been determined explicitly in several neurodegenerative disorders. These molecules act by modulating the acute processes involved in functioning of the brain by enhancing neurogenesis, and hence impose a potent beneficial role in management of neurological disorders. The endocannabinoid system has been studied to decrease the instances of neuroinflammation and prevent the detrimental effects that led to new row degradation by supporting pathways that encourage natural repair and protection of neurons. The endocannabinoids present neuroprotective action by encouraging neurogenesis and preventing neuroinflammation and neurotoxicity [16,93].

### 5.1. Endocannabinoids and Neurogenesis

The cannabinoid molecules have shown promising activity in regulating the pathways associated with neurogenesis. The progenitor cells in the hippocampal region in adults, and the embryonic cells, produce endocannabinoids, which are mainly expressed upon stimulation of CB1 receptors and suppression of FAAH enzyme [94]. Proliferation of neuronal cells and generation of neurospheres is promoted by activation of CB1 receptor. A study with mice deficient in FAAH enzyme showed an increase in neural progenitors, which promoted proliferation of hippocampal neurons and was vice versa decreased in mice with knocked out or decreased CB1 receptors. The meticulous function of CB1 receptors in neurogenic pathways is still unclear; however, several studies have signaled that the proliferation of cells was increased, and differentiation of neurons was decreased in its absence [94]. The antidepressant and anxiolytic effect of HU210, a potent agonist of CB1 receptor, suggests a functional role of CB1 receptors in neurogenesis in the hippocampal region of brain. The CB1 receptors are misplaced from the areas of basal ganglia during early onset of neurological disease, and hence indicate the presence of these receptors in proliferation of neuronal cells [95]. Evidence collected to date suggests that the cannabinoid receptors propel cell division by the activity of MAPK/ERK and phosphoinositide 3-kinase (PI3K/Akt) pathway chiefly through CB2 receptors. ACEA, a CB1 agonist, might inhibit phosphorylation of ERK in the progenitor cells of neurons while HU-308, a CB2 agonist, increases proliferation of neural progenitor cells via both the PI3K/Akt and ERK pathway. The cannabinoid receptors, upon activating various pathways in the neural progenitor cells, convert into transcription factors like cAMP, which further leads to element-binding proteins, T-box transcription factor (Tbr), and the Sox2 family. The latter acts upon stimulation of CB1 receptors and via notch signaling [96,97]. The endocannabinoid system interacts with the epidermal growth factor receptors and regulates the growth and division of neural stem cells via both CB1 and CB2 receptors in the brain. Many other growth factors like brain derived neurotrophic factor (BDNF), protein kinase C, and interleukin 1 also interact with cannabinoid receptors and can be targeted as a therapeutic approach in the management of neurological diseases.

### 5.2. Endocannabinoids and Neuroprotection

The cannabinoids possess neuroprotective roles via their extensive linking to the inflammatory and excitatory nonreceptor-mediated pathways. They have shown proven protection against conditions of acute hypoxia, oxidative stress, traumatic insults, and excitotoxicity. The levels of endocannabinoid 2-AG were dramatically elevated in mice with a closed head injury. Further administration of 2-AG exogenously diminished edema in the brain, decreased the infarct volume, prevented death of hippocampal neuronal cells, and facilitated a better clinical recovery [98]. The loss of hippocampal neurons and volume of the cerebral infarct in the rat model of traumatic brain injury using focal cerebral ischemia was significantly decreased upon administration of WIN55,212-2, a cannabinoid agonist. BAY 398-7271, an agonist of cannabinoid receptors, presented potent neuro protective properties in similar models of brain injury. Several endocannabinoid mimetic agents are currently being investigated for neuroprotective activity in ischemia and traumatic brain injuries, and are under clinical trials. The formation of endocannabinoids is dependent upon the concentration of calcium ions and can be applied as a feedback inhibition during conditions of excitotoxicity. The release of serotonin, gamma GABA, noradrenaline, acetylcholine, and other neurotransmitters is inhibited upon activation of CB1 receptors depending upon the type of neurons in which the receptors are expressed [99,100]. The rate of activity of these neurotransmitters can be suppressed by their retrograde inhibition at the postsynaptic neuronal site. The endocannabinoids which are released from the post synaptic neurons disseminate through the synapse to bind with the CB1 receptors present at the presynaptic site and decrease the fusion of vesicles with membrane thereby shrinking cAMP concentrations and further modulating phosphorylation mediated by protein kinase A and MAPK pathway. The decreased cAMP levels further inactivate the opening of calcium channels and thereby have a direct effect on the A-type potassium channels and reduce neuronal excitation. Therefore, endocannabinoids have been described to play a neuroprotective role and can be explored as a potent therapeutic approach in the management of chronic neurological diseases [101,102].

### 5.3. Endocannabinoids and Parkinson’s Disease

Parkinson’s disease is a late onset chronic progressive neurodegenerative disease characterized by symptoms of involuntary motor tremors, rigidity in muscles, unstable posture, abnormal gait, and akinesia. The disease is typically idiopathic and is also genetically associated. The substantia nigra parts compacta region of the brain is mainly affected in this disease, with over 70 to 80% of occurrence of neuropathological atrophy leading to extensive loss of dopaminergic neurons even before the diagnosis and onset of clinical symptoms of the disease [103]. The dopaminergic neurons in the region of substantia nigra pars compacta are vital for signaling of the chief neurotransmitter dopamine, which projects motor activity. Mitochondrial defects, increased free radicals-medicated oxidative stress, and activation of glutamate are some of the dysfunctional mechanisms that lead to neurotoxicity and result in onset of this debilitating neurological disorder. The primary target of therapies in management of the disease include replacement of dopamine for symptomatic control and no treatments are available that directly slow down the progression of degeneration in the nigral region. The pathology of Parkinson’s disease includes hyper functionality of the cannabinoid receptors [104]. The levels of anandamide were detected to be almost double in the cerebrospinal fluid of an untreated patient with Parkinson’s disease in comparison to healthy individuals, independent of the stage of disease, concentration of drug, or range of symptoms experienced by the patient [105].

### 5.4. Endocannabinoids and Anxiety

Anxiety affects over more than 40 million people alone in United States and is an important condition for research as anti-anxiety drugs are among the topmost prescription drugs sold in the market. Cannabis has been extensively used since millennial times as a medicinal agent and is believed to help the consumer and produce soothing effects. However, direct data from experimental studies on exploration of endocannabinoids in patients with anxiety is still limited. The several physiological actions of endocannabinoids and their major role in release of neurotransmitters apparently suggests their effect in management of anxiety, which is an outburst of stressful conditions in humans. An anxiolytic effect of FAAH Enzyme inhibitors, namely URB532 and URB597, has been detected in rats undergoing an elevated zero maze test due to elevation of anandamide levels in the brain in lieu of blocking the activity of CB1 receptors. This led to the confirmation of anxiolytic activity of anandamide [106]. The evidence supporting this activity is the production, release, and mechanism of action of anandamide since it is not stored in the synaptic vesicles of the neurons but released upon synthesis in the synaptic cleft post stimulation. Upon blockage by URB597, CB1 receptors were found to induce anxiogenic like behavior, indicating the possible role of endocannabinoid receptors as a potent therapy in management of anxiety. The similar effects were also noted upon augmenting the concentrations of 2-AG, which further stimulated the signaling of endocannabinoid receptors [107]. A suitable role of CB2 receptor besides CB1 receptors was evaluated in the management of anxiety as anxious mice comprising of overexpressed CB2 receptors presented reduced anxiety-like behavior, indicating its direct involvement in modifying the responses to conditions of stress.

### 5.5. Endocannabinoids and Depression

**Figure 5:**
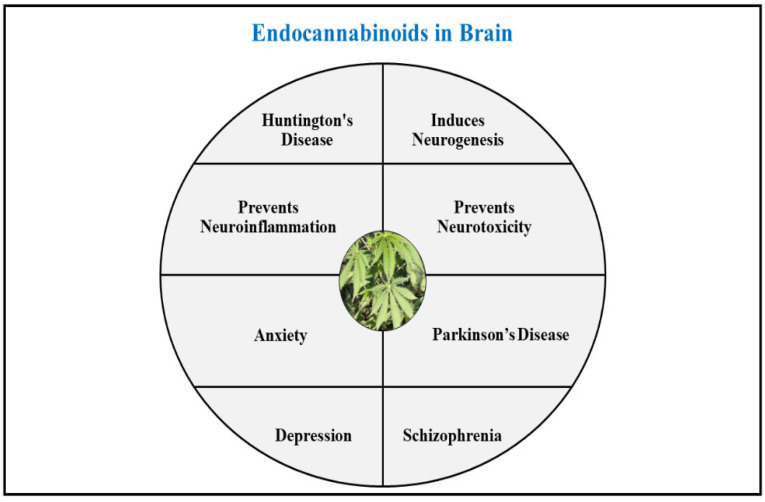
A general overview of the beneficial roles presented by endocannabinoids in brain.

Several studies performed to date have established the presence of depressive symptoms under lower concentrations of endocannabinoids, and anti-depressant effects upon higher levels of endocannabinoids, supporting the evident role of endocannabinoids in management of depression. The hypothesis supporting this activity is the considerable neurogenic effects produced by endocannabinoids since the process of neurogenesis is downregulated in depressive patients [108,109,110]. Upon activation, the CB1 receptors induce proliferation of progenitor cells and endorse neurogenesis. Mice with knocked out CB1 and CB2 receptors demonstrated reduced neurogenesis. 2-AG and the enzyme involved in its synthesis diacylglycerol lipase (DAGLs) stimulate proliferation and growth of axons and stimulate CB2 receptors, which causes further promotion of proliferation of neuronal progenitor cells. Therefore, synthesis of endocannabinoid-like compounds has also been suggested as a novel anti-depressant activity, and extensive research on development of the same is being performed [25,111,112] (Figure 5).

## 6. Conclusions

Research into cannabis was initially initiated with a limited aim of understanding the action of this illicit drug. After thorough research on the chemistry of the plant and its logical and psychological activities, the compound THC was isolated. This research began most intently in 1960s and early 1970s. By the mid-1980s, two major specific receptors and their associated ligands linked with the endocannabinoid system were identified, and their biological processes showed a wide spectrum of clinical implications. The discovery of the endocrine system has opened wide areas of research in life sciences, particularly in the area of the central nervous system. The main action initiated upon activation of the cannabinoid receptors chiefly CB1 receptors leads to inhibition of neurotransmitter release. In lieu of this activity, the endocannabinoids are believed to reduce excitation of presynaptic neurons and are responsible for the major renowned marijuana effects, including anxiety and cognition impairment. Immunosuppression is also a major consequence of activation of cannabinoid CB2 receptors, which helps to reduce inflammation and prevents associated injury in tissues. The enhancement of expression of CB2 receptors has been targeted to cure numerous diseases, and has generated a protective effect in a wide array of physiological systems. The current review summarizes the physiological roles attained by endocannabinoids and their relevance in treatment and management of several chronic diseases.

## Figures and Tables

**Table 1:** The below table lists examples of the most commonly used compounds that act via endocannabinoid receptors and initiate numerous physiological responses.

S. No.	Name of Compound	Activity/Mechanism of Action
1.	HU-210	CB1 Receptor Agonist
2.	Δ9-THC	Partial Agonist of CB1 and CB2 Receptors
3.	HU-308	Selective CB2 Receptor
4.	CP-55940	Potent and Complete Agonist of CB1 and CB2 Receptors
5.	R-(+)-WIN-55, 2/2-2	Complete CB1 and CB2 Agonist; Affinity Higher towards CB2 Receptor
6.	Anandamide	Partial CB1 and CB2 Agonist
7.	2-AG	Complete CB1 and CB2 Agonist
8.	Arachidonyl-2′-chloroethylamide (ACEA)	CB1 Agonist
9.	SR 141716A	CB1 Receptor Antagonist
10.	SR 144528	CB2 Receptor Antagonist
11.	LY320135	CB1 Receptor Antagonist
12.	AM 630	CB2 Receptor Antagonist; Low Affinity CB1 Partial Agonist
13.	LH-21	CB1 Receptor Antagonist
14.	AM 404	Transport Inhibitor
15.	VCM 707	Transport Inhibitor
16.	AM 1172	FAAH-Resistant Transport Inhibitor

## Data Availability

Not applicable.
